# Management Modalities of Mandible Fracture and Dental Trauma in Pediatric Patients During Mixed Dentition Phase: A Series of Case Study

**DOI:** 10.1155/crid/6662463

**Published:** 2025-01-02

**Authors:** Lomtu Ronrang, Alice Lyngdoh

**Affiliations:** ^1^ Department of Dentistry, NEIGRIHMS (North Eastern Indira Gandhi Regional Institute of Health and Medical Sciences), Shillong, India

**Keywords:** dental trauma, noninvasive management, clinical outcome

## Abstract

The prevalence of oromaxillofacial fracture in pediatric patients is comparatively less than in adults, which could be due to several inconclusive factors, such as infrequent exposure to high‐contact sports games, rash driving of vehicles and motorbikes, alcohol consumption, and fist fights for personal reasons under the influence of alcohol. More importantly, most of the time, children are under the care of their parents till they reach an age of maturity. One more thing that everyone believes even today is the elasticity nature of their bones as well as their body weight during their growing stage. All these uncertain factors, including the natural way of protection bestowed on us by nature during the childhood stage, might be the actual factors that can easily resist any form of minor trauma in pediatric patients. But sometimes, we come across children who have suffered traumatic head and neck injuries, including mandible fractures, usually falling from a height, and road site accidents while playing on the roadsides, including traffic accidents, which are inevitable. This is a case of three pediatric patients who had experienced accidental dental trauma with slight‐displaced mandible fractures with slight dearrangement of normal occlusion and avulsion of permanent teeth. The cases were managed individually according to the types of trauma and injury they had sustained. Among three cases, one was a 9‐year‐old female pediatric patient, and the other two were male pediatric patients of 7 years old. Clinically, female patients had no mandible fracture as such except avulsion of teeth in the region of 32, 33, and 34. Both the male patients had a slight displacement of the mandible with a dearrangement of occlusion, and one male patient had a bilateral mandible fracture. So considering the nature and type of an injury they had sustained individually, different treatment modalities were employed for each individual as per their requirement for the restoration of normal occlusion, including hard and soft tissues. The prime objectives were the restoration of normal occlusion and alignment of the jawbone, including esthetic and phonetic, without impairing normal growth and development of jawbone and permanent tooth buds. The purpose of this article is to emphasize noninvasive methods of pediatric mandibular fracture reduction, restoration of normal occlusion, and management of soft tissue. Our treatment modalities show that this method can be easily applied in cases of any slightly displaced mandibular fracture in children during mixed dentition periods while taking utmost care of permanent tooth buds compared to other invasive methods of pediatric mandibular fracture management. The application of these various noninvasive treatment modalities and their clinical application for the management of slight displacement of mandibular fractures in pediatric cases were effective and clinically apparent in our study. The study also shows that various treatment modalities employed for the management were acceptable in terms of patient compliance due to noninvasive methods of management for slightly undisplaced mandibular fractures in pediatric cases.

## 1. Introduction

Fracture of the facial skeleton occurs infrequently in children when compared with the prevalence in adults [1]. So far, no concrete theories are present on which we can rely for the low prevalence of fractures in children. However, as per [2], the prevalence and etiology of pediatric facial fractures vary somewhat from study to study depending on the age grouping used by the authors, the location of the reporting institution, and the population it serves. In European countries, the prevalence in children younger than 12 years ranges from 1.5% to 8% of all facial fractures (adults and children) in trauma centers [2]. In children younger than 5 years, the reported prevalence is approximately 1% of all facial fractures [3]. In Africa, Chidzonga reported an incidence of 3.3% in Zimbabwean children, while Adekeye recorded 0.5% for children 5 years and below in Northwest Nigeria [4, 5]. The most common cause of facial fractures in children in Australia is due to falls [6], while violence has been reported as the single most etiological factor in South Africans under 18 years of age [7]. In the central part of India, their [8] study has shown that the prevalence rate of mandibular fractures among different age groups seems to be 4.5% in 0–10 years of age and 17.8% in the 11–20 year age group.

As per their data, fractures of the mandibular according to etiology have been found to be 68% in road traffic accident (RTA), 11% with assault, 17% due to falls, and 4% miscellaneous. It has also been said that the highest incidence of mandibular fractures is found in the age group of 21–31 years (37.5%), with more male dominance in gender distribution, which was around 3.7:1 male to female ratio, as compared to the other study, which was around 5.1:1 male to female ratio [9]. The clinical cases of our study are also the same as the other cases in children’s trauma, with almost the same mechanisms of management in every case. However, the mechanism of management pertaining to those children, especially in case two, appears to be very much acceptable in terms of patient compliance, demonstrating a tangible clinical outcome with our methods of management.

## 2. Report on Series of Cases

This is a series of clinical case studies in three pediatric patients, comprising two boys and one girl ages 7, 7, and 9 years old. Clinically, the types of injury sustained were different individually, like slight displacement of mandible bone due to fracture and dearrangement of normal occlusion in two male pediatric cases, with one male patient having a bilateral mandible fracture. Avulsion of teeth was found in a female pediatric patient during an intraoral examination. All three patients had the same history of accidental trauma by falling on the roadside while playing. The two male patients were referred to the dental outpatient department (OPD), North Eastern Indira Gandhi Regional Institute of Health and Medical Sciences (NEIGRIHMS), Meghalaya, India, following a traumatic injury from an emergency and pediatric department. The two male pediatric patients received premedication and other necessary emergency management from the concerned pediatric and emergency departments before they were referred to dental OPD. Only a female patient was managed right from the time of emergency till the delivery of removable partial denture (RPD) and plastic surgery for the scar. All these were managed during the period from October 2019 to January 2023, especially in Case I.

### 2.1. Case No. 1

**Figure 1:**
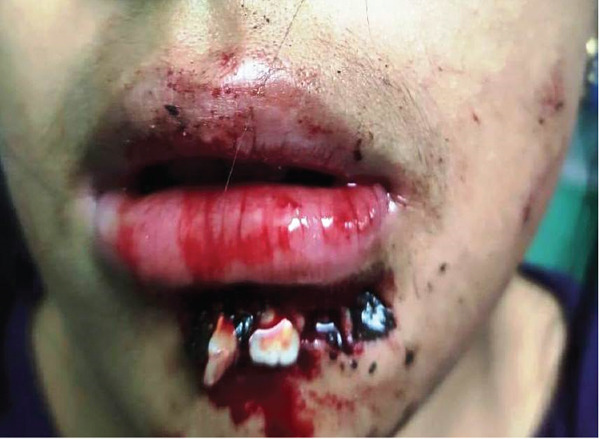
Front view of avulsion of Tooth Nos. 32, 33, and 34 due to accidental fall.

**Figure 2:**
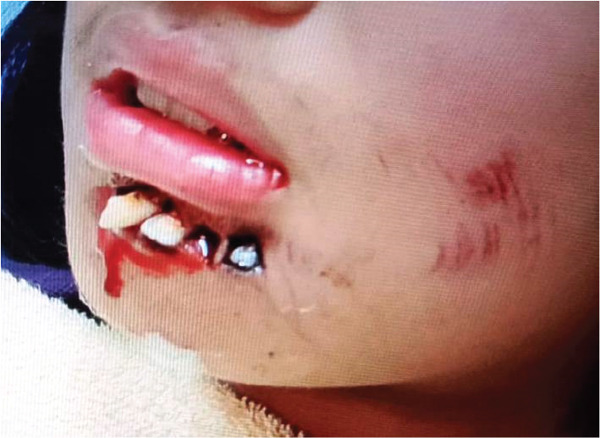
Side view of Tooth Avulsion Nos. 32, 33, and 34 due to accidental fall.

**Figure 3:**
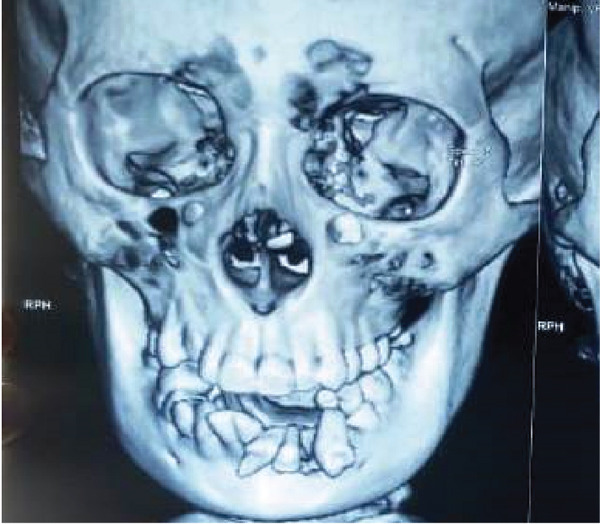
Front view of 3D x‐ray shows avulsion of teeth in the region of 32, 33, and 34.

**Figure 4:**
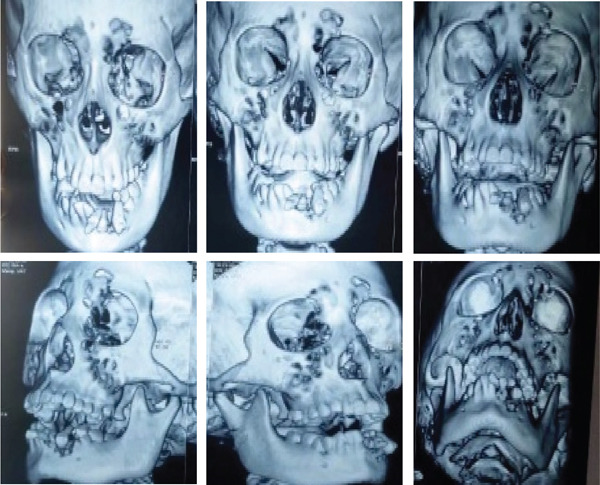
Different angles of 3D x‐ray show different regions of the mandible and condyle with avulsion of teeth in the region of 32, 33, and 34.

**Figure 5:**
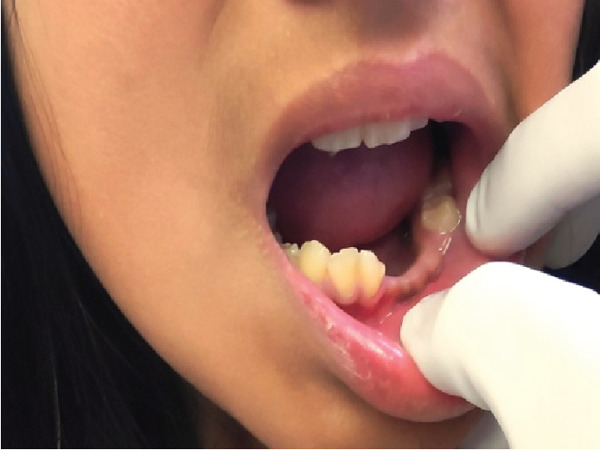
After 1 month postmanagement shows healing of edentulous area in a region of 32, 33, and 34 tooth nos.

**Figure 6:**
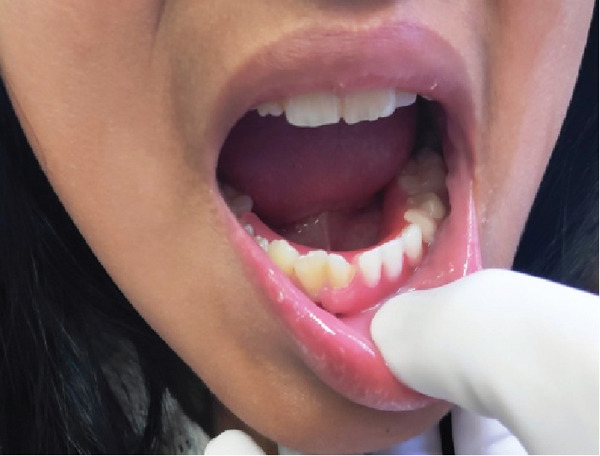
After fabrication of flexible removable partial denture in a region of the edentulous area.

**Figure 7:**
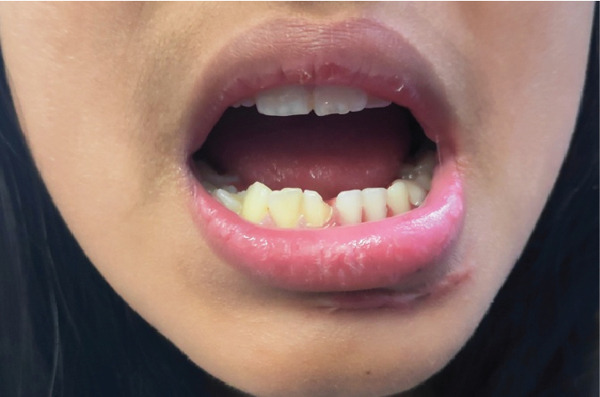
Scar below a lower lip.

On intraoral examination and clinical examination in Figures 1 and 2, the patient had only dental trauma with avulsion of Tooth Nos. 32, 33, and 34. No sublingual ecchymoses or any other clinical signs of jaw fracture were observed. The patient was sent for a 3D x‐ray of the oromaxillofacial region for further evaluation of facial bone structure (Figures 3 and 4). After observing the x‐ray, all avulsed teeth were removed under local anesthesia. The mucosa was sutured with 3–0 black silk, and the skin was sutured with 5–0 monofilament (nylon). A prophylactic antibiotic was prescribed according to the child’s body weight for 7 days, and an analgesic was prescribed for 3 days following the recommended dosage based on the child’s body weight. Proper postoperative oral hygiene instructions, including dietary advice, were provided. The patient was reviewed after 7 days, and the sutures were removed. Since she is a girl child and still growing, she does not feel comfortable attending any social activities without teeth. To make her comfortable, we planned for the fabrication of flexible RPDs after 1 month since there is no alternative apart from a RPD. Flexible RPD was fabricated following the alginate impression, and RPD was delivered to her for the restoration of esthetics, phonetic, and functional purposes of the edentulous area (Figures 5 and 6). Proper planning of partial removable dentures for a patient was done as per the growth and development of a child. The design of dentures must allow for modification when teeth erupt or migrate. It is said that long periods without a tooth (or tooth replacement) lead to the narrowing of alveolar processes and vertical alveolar defects at sites with missing teeth, overeruption of unopposed permanent teeth, and tipping of adjacent teeth [10]. Some studies also mention that premature teeth loss, both deciduous and permanent, may cause functional problems in children, such as malfunctions in mastication, improper tooth placement or eruption, and hindered pronunciation. Esthetical issues are also present, as children may be mocked or bullied, leading to insecurity, the development of complexes, and low self‐esteem [11]. As the child was growing, within 2 years of time, the denture was not fitting, which made it difficult to fit and wear it anymore. The new flexible RPD was replaced after 2 years (Figure 7), and now the patient is wearing a new flexible RPD.

**Figure 8:**
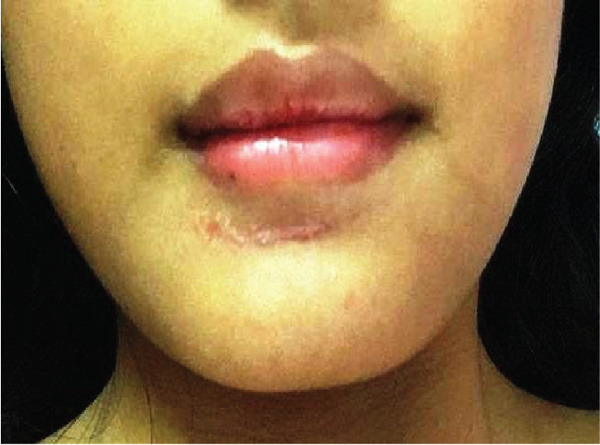
Reduced in size of scar after plastic surgery.

Some studies also say that prosthetic appliances need to be regularly maintained, adjusted, and checked in order to prevent them from inhibiting proper orofacial development [11]. Finally, patients want to go for the removal of a scar for cosmetic reasons and have undergone plastic surgery to reduce the visibility of a scar (Figure 8). This process continued for almost more than 4 years till the patient underwent plastic surgery to restore facial esthetic impairment.

### 2.2. Case No. 2

**Figure 9:**
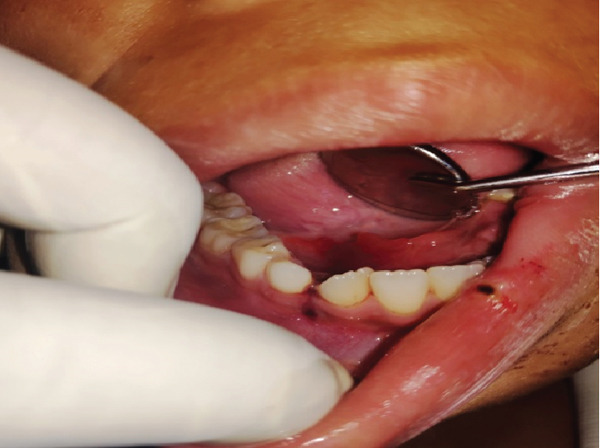
Ecchymosed in the lower lingual right side of 83 and 84 region of oral cavity.

**Figure 10:**
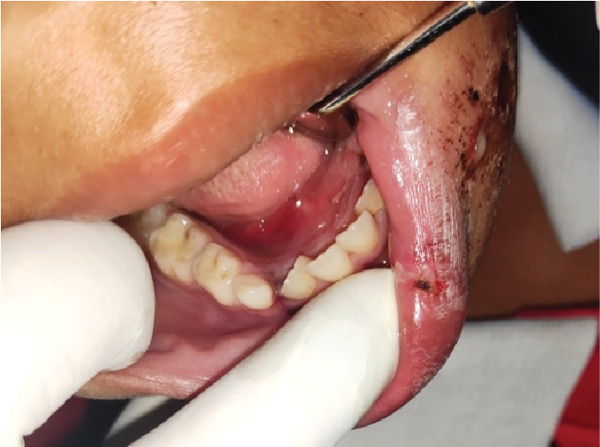
Examining the fracture area of the mandible in the region of 83 and 84 of teeth in a mandible.

**Figure 11:**
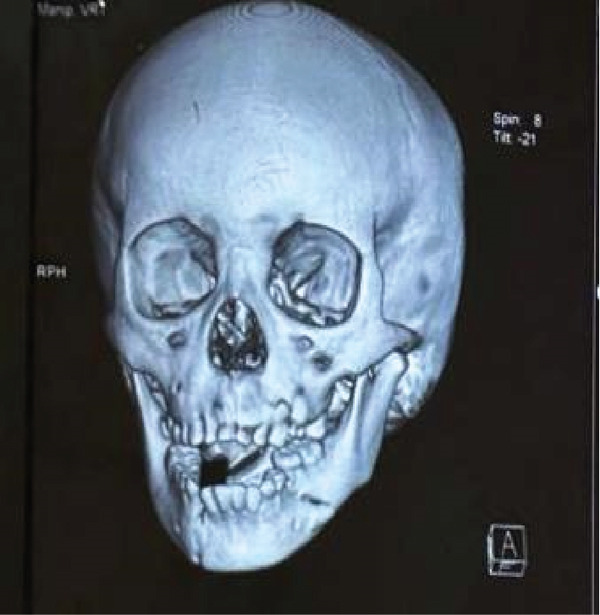
3D x‐ray shows bilateral parasymphyseal fracture of the mandible—front view.

**Figure 12:**
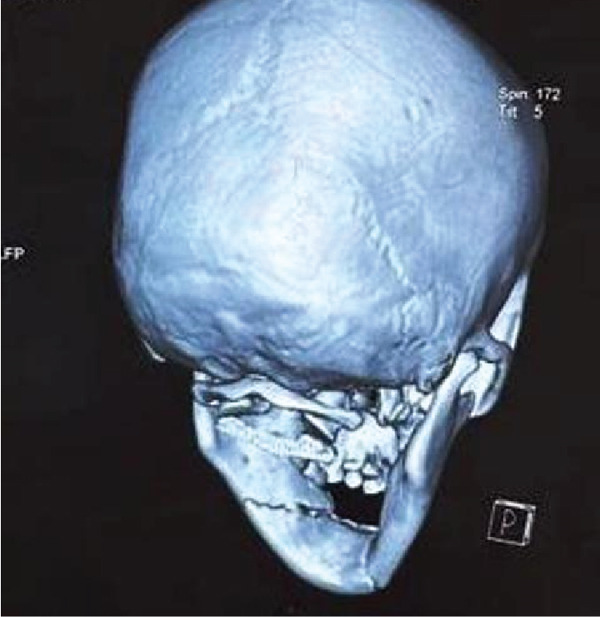
3D x‐ray shows bilateral parasymphyseal fracture of the mandible—a posterior view.

**Figure 13:**
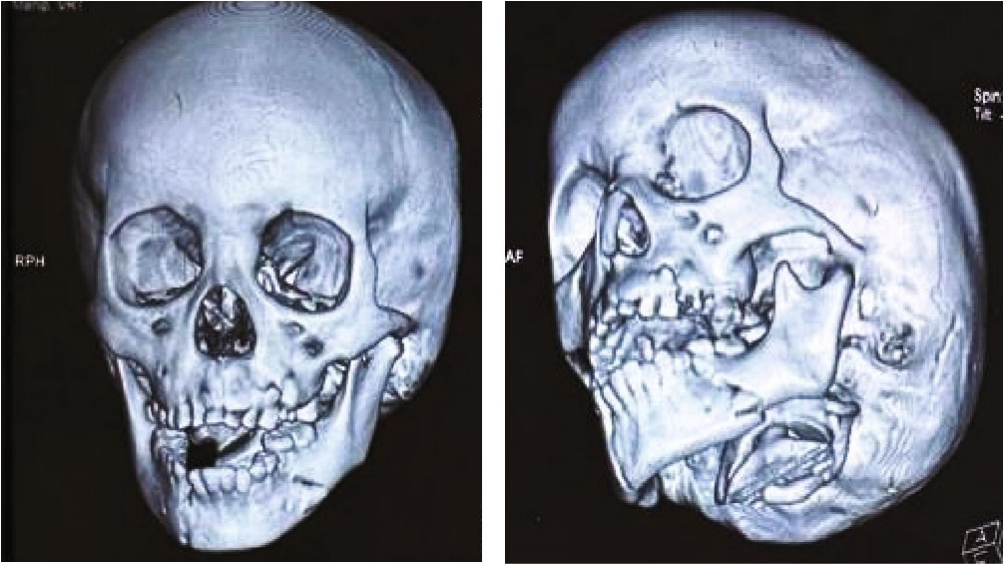
3D x‐ray shows bilateral parasymphyseal fracture of the mandible with open bite—front and side view.

**Figure 14:**
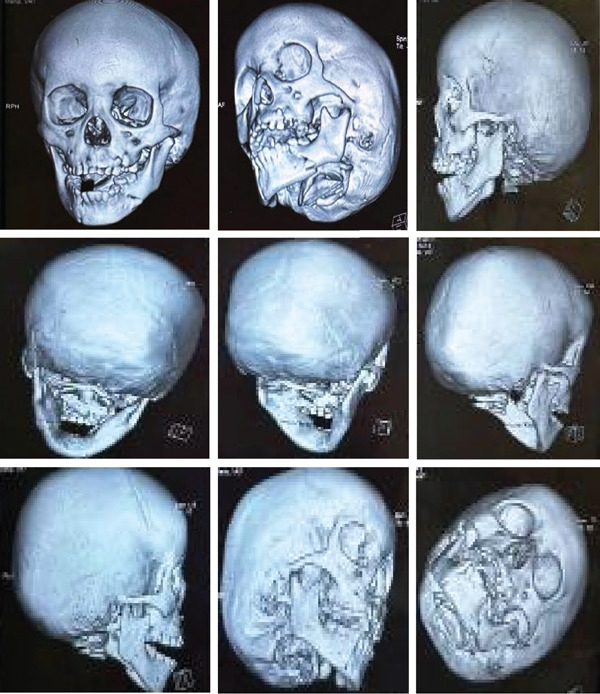
3D x‐ray shows bilateral parasymphyseal fracture of the mandible with open bite in different angles.

This 7‐year‐old male patient had a bilateral mandibular fracture that had been referred from the pediatric department with a history of accidental falls (Figures 9 and 10). Clinically, there was slight dearrangement of occlusion with an open bite and abrasion of skin on the accidental side; no intraoral ecchymosis was found as such. After observing the 3D x‐ray of the patient, it shows a bilateral fracture of the mandible as shown in Figures 11, 12, 13, and 14.

**Figure 15:**
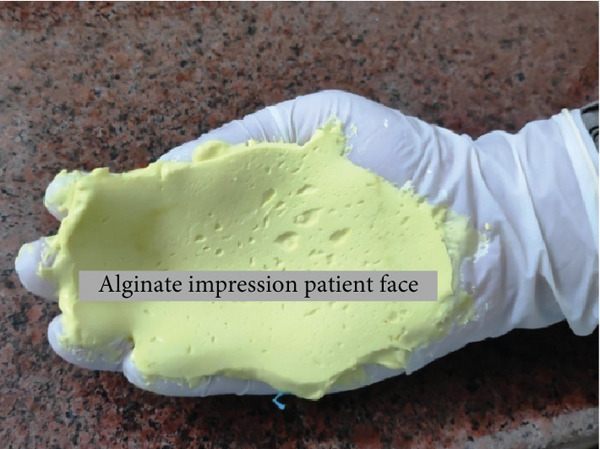
Procedure of an alginate impression with the hand (palm) of both sides of the face with the same procedure.

**Figure 16:**
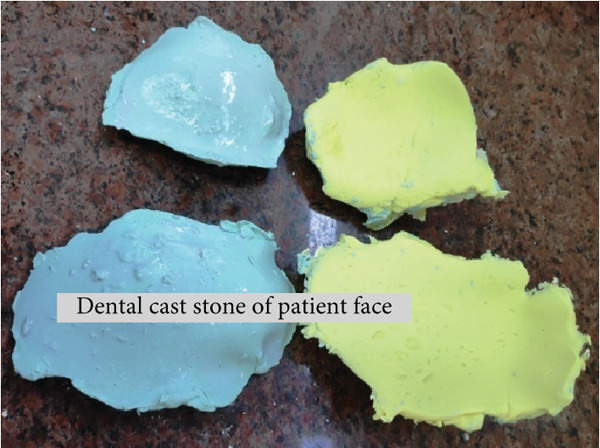
Alginate impression taken with hand (palm) of both the right and left side of the patient’s face and dental cast stone for fabrication of an acrylic splint.

**Figure 17:**
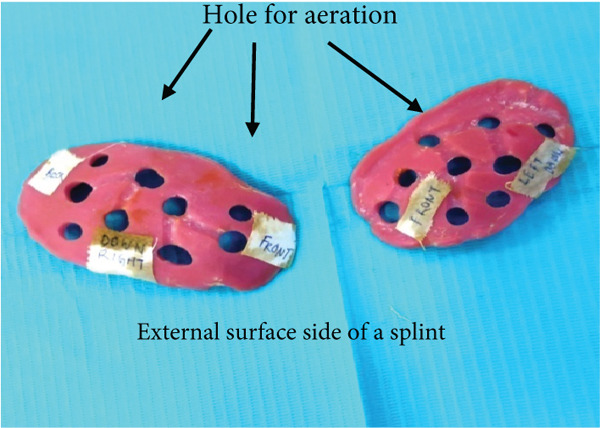
External surface of customised acrylic splint with holes for the aeration and for the fixation of moldable orthodontic wire to an attached elastic.

**Figure 18:**
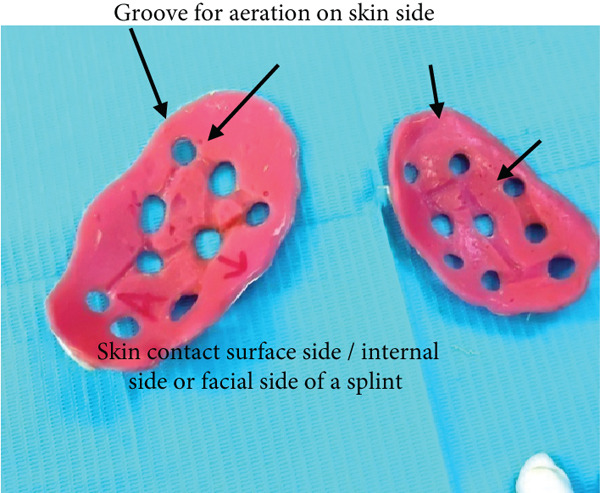
Customized acrylic splint with holes and grooves for aeration as well as for lightweight.

As the patient has bilateral cross‐bite in occlusion, it needs to be restored to normal occlusion as much as possible. But treatment principles for pediatric mandibular fracture differ from those of the adult population in that a conservative approach is advocated in most cases [12]. Even they said that open reduction and osteosynthesis of the pediatric fracture with titanium plates and screws are thought to have a negative effect on skeletal growth and unerupted teeth [13]. It also involves two‐stage surgery because of the need for plate removal after complete healing [14]. Having considered the shortcomings of various invasive approaches for the reduction of mandibular fracture, conservative management is the only option for this case. We took an alginate impression on both sites of a patient’s face after the application of Vaseline on the impression area of the patient’s face. Impression is taken with the help of the right hand (palm), which acts as an apparatus for holding impression materials on the face till they are set (Figure 15). Make sure that the impression should extend to the required area as per the requirement and the needs for fabrication of the appliance, which will function effectively during the application of the customised fabricated appliance. The same procedures were followed for the other side of the face. Once an alginate impression is done, a dental cast stone is poured (Figure 16) on both sides of the impression, and the casts will resemble a patient face. Now fabricate a splint with self‐cure acrylic resin (polymethyl methacrylate (PMMA)) the way we manipulate during the repair of a denture on dental stone casts after application of a cold mould seal. A splint should be made on the cast of the patient’s facial side of the impression till the desired thickness and extension are achieved and allow it to set. Once the cold‐cured acrylic resin is set, it is trimmed as per the requirement with the help of an acrylic bur trimmer, which will act as a splint. The right side of the splint had a bit more extension than the left side since this side required more alignment than the contralateral side. Holes were made at the edge of a splint and most parts of the area to provide aeration and for attaching wire and elastic bands (Figures 17 and 18). Grooves were also made on the facial skin side of a splint as well as on the outer side of a splint since most of the time facial skin will remain covered or in contact with an acrylic splint during the course of application. These grooves will provide aeration along with a reduction of unnecessary weight and thickness of a splint without compromising its functional effectiveness during application.

**Figure 19:**
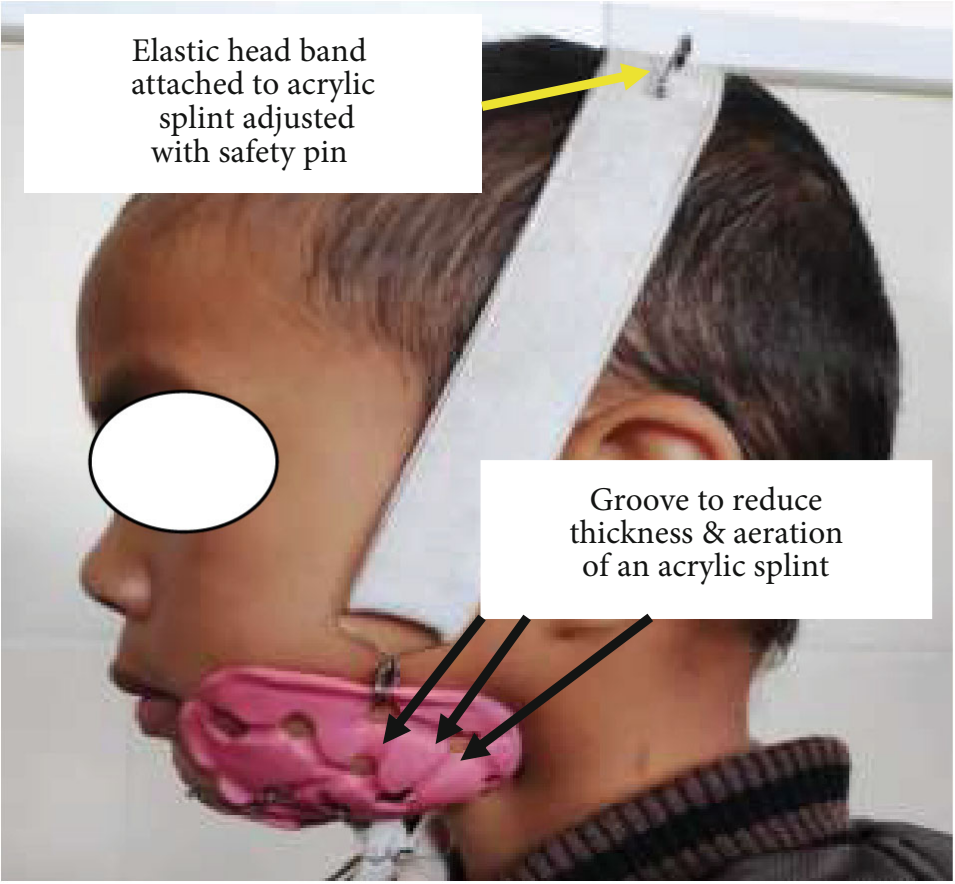
An elastic band attached to customized acrylic splint with moldable orthodontic wire—left side.

**Figure 20:**
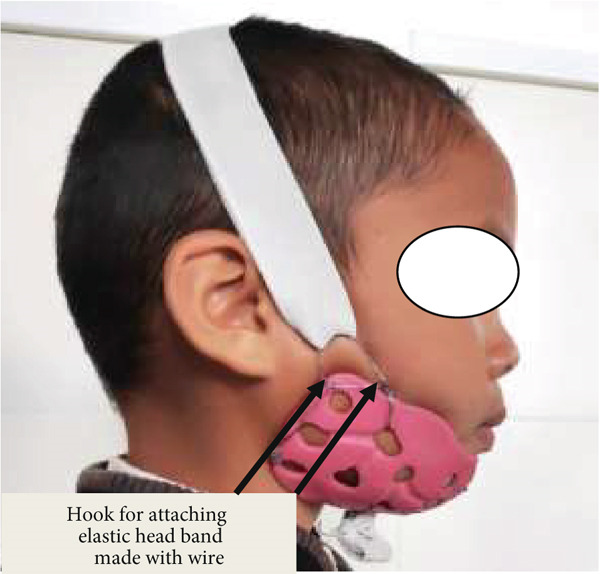
An elastic band attached to customized acrylic splint with moldable orthodontic wire—right side.

The contralateral side was also fabricated with the same procedures. After completion of trimming and polishing the customised acrylic resin splint, orthodontic wire with 26 gauge was rolled together and made a hook for the attachment of an elastic band, and the appliance was made ready (Figures 19 and 20). During the time of delivery, the splint and elastic band were adjusted according to the need for elastic pressure required for the correction and stabilization of a deviated jaw and occlusion. In the initial stage of appliance application, patients were advised to review after every week to check the effectiveness and efficiency of an appliance as well as any adverse effects of the splint pressure.

During the initial stage of treatment, patient guardians were instructed to use a crepe bandage (Figure 21(a)) along with a customised acrylic splint. Both the acrylic splints were joined with moldable orthodontic wire (Figure 21(b)).

Figure 21(a, b) Crepe bandage application during the initial phase or active phase of treatment.

**Figure:**
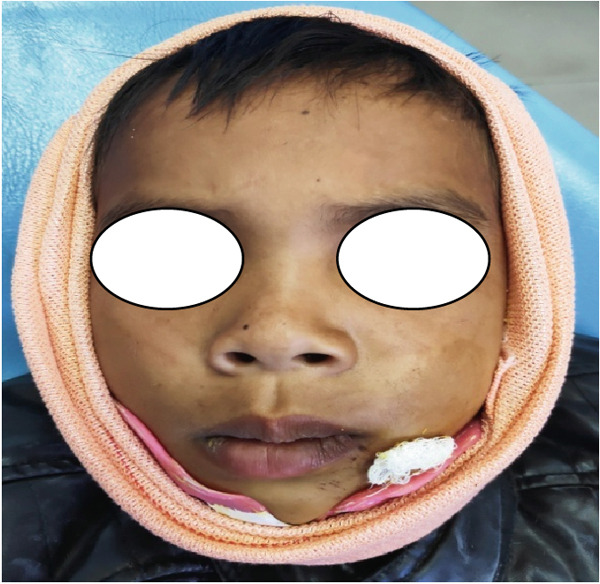
(a)

**Figure:**
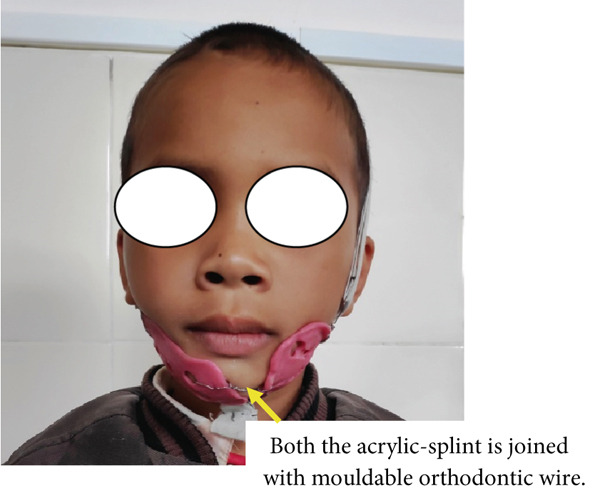
(b)

**Figure 22:**
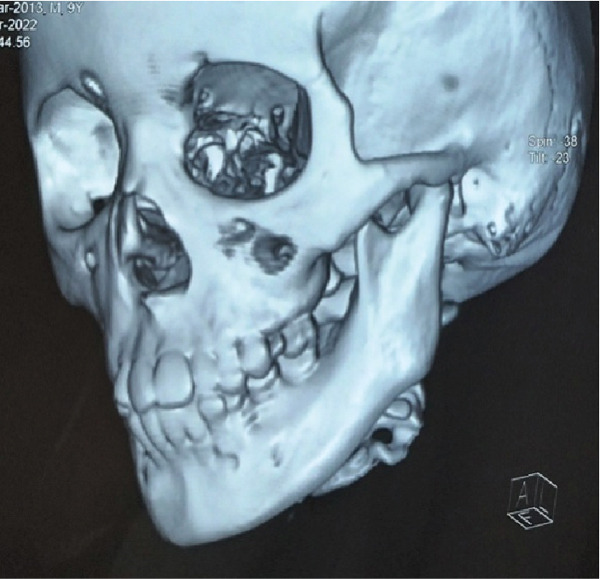
3D x‐ray view of postmanagement of the mandible fractures healed without derangement in occlusion.

**Figure 23:**
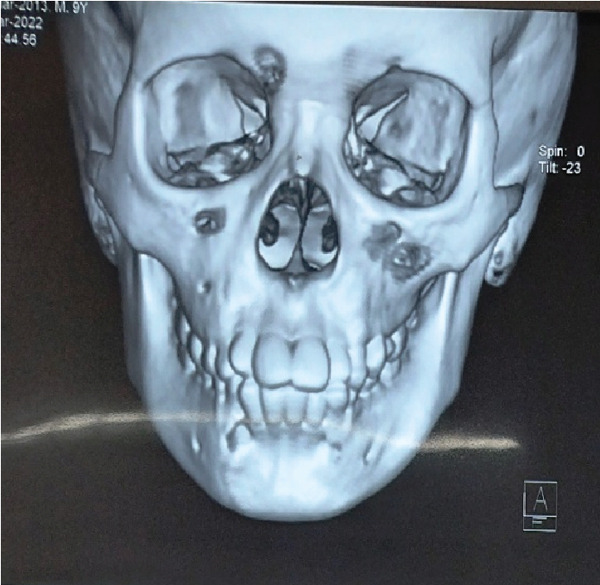
3D x‐ray view of postmanagement of the mandible fractures healed without open bite in occlusion—front view.

**Figure 24:**
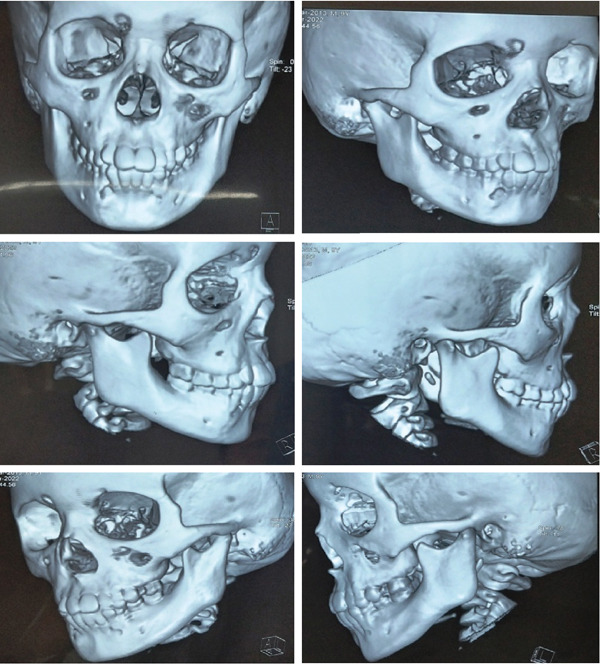
3D x‐ray view of postmanagement in different sides of the mandible fractures healed without dearrangement in occlusion.

**Figure 25:**
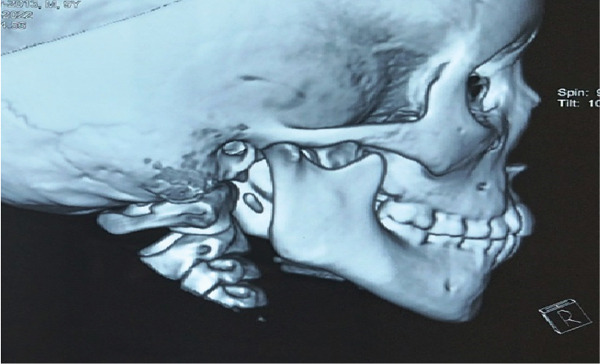
3D x‐ray view of postmanagement shows both sides of mandible fractures healed without dearrangement in occlusion.

It was also instructed that excessive compression should be avoided, which might cause inconvenience to a patient. A patient’s parent was instructed on a soft diet or semisolid diet for at least 3–4 weeks and advised to review as per our instruction for follow‐up. The process of adjustment and modification of an elastic band and splint was followed for about 1–3 weeks till the patient was accustomed to it. The patient was advised to wear it during the daytime as much as possible, except during eating and sleeping at night. During sleeping, the patient was advised to adjunct with a crepe bandage instead of a splint. This procedure continues for almost 3–4 weeks. As the patient started improving, the patient was rescheduled to review every 15 to 1 month, followed by 2 months, 6 months, and once a year for almost 2 years. Once the patient had recovered to normal occlusion, we advised doing 3D x‐rays of the jaw to see the present status of the mandibular jaw fracture (Figures 22, 23, 24, and 25), which show proper alignment of the jawbone as well as restoration of normal occlusion clinically.

### 2.3. Case No. 3

**Figure 26:**
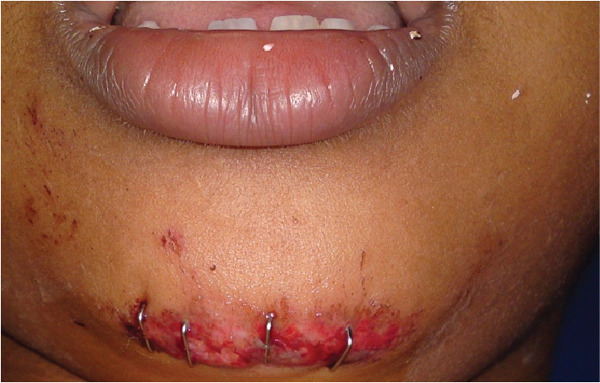
Traumatic fall on the road while playing, suture a wound with staple pins.

**Figure 27:**
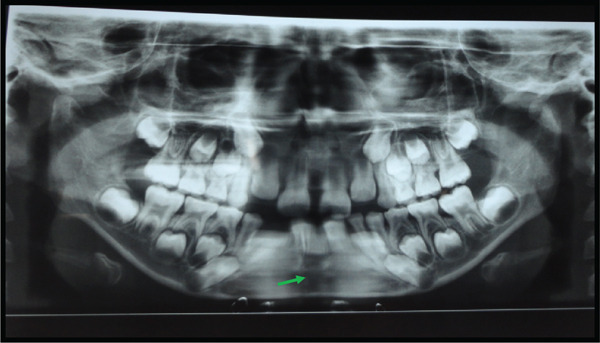
Orthopantomograph of the jaw.

**Figure 29:**
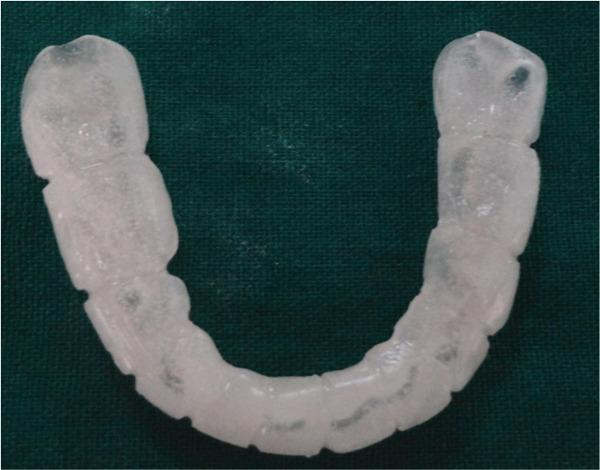
A fabricated a cap splint to stabilized the fracture of the jaw.

**Figure 30:**
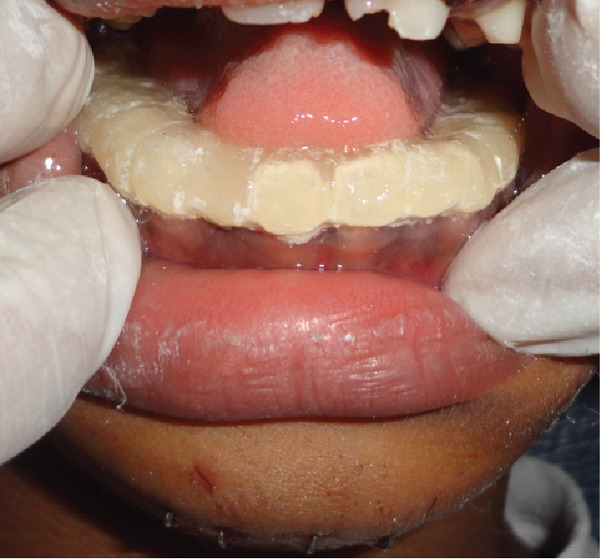
Cap splint and its application in the arch of the teeth to stabilize the fracture of the jaw.

**Figure 31:**
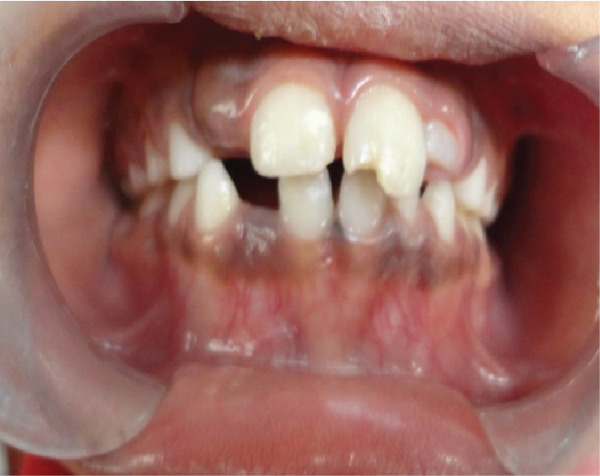
Postmanagement of fracture in the anterior lingual region after cap splint.

This is a case of a male pediatric case with age 7 years old who had reported to dental OPD with a history of accidental falls while playing on the road. Preliminary management was already done in the emergency department before attending dental OPD. The wound was closed with a stapler pin, as shown in Figure 26. The only concern was for an undisplaced mandible fracture seen in an orthopantomograph and other x‐rays of the jaw (Figures 27 and 28(a)). On clinical examination, lingual ecchymosis is found in Figure 28(b), and a slight dearrangement of occlusion was present with slight difficulty in chewing food. According to some studies, many pediatric jaw fractures are nondisplaced or greenstick‐type fractures, and observation alone is adequate. A conservative approach (observation or closed reduction) is the best approach to consider first for pediatric mandible fractures, as these fractures heal rapidly and the children grow normally [15, 16]. However, this case had some difficulty chewing, which required stabilizing an undisplaced fracture of the mandible, so a cap splint was opted for and planned for this case. As per them, a cap splint provides close reduction and stabilization of mandibular fracture and allows hygiene maintenance without disturbing tooth buds [17]. Several studies have also recommended the use of prefabricated acrylic splints as a treatment for pediatric mandibular fractures. These splints are more reliable than open reduction or intermaxillary fixation (IMF) techniques with regard to cost‐effectiveness, ease of application and removal, reduced operating time, maximum stability during the healing period, minimal trauma for adjacent anatomical structures, and comfort for young patients [18]. So the patient in this present case was managed with a custom‐made cap‐splint fixed with luting cement, which was fabricated on dental stone casts after taking an impression of the lower arch (Figures 29 and 30). The patient was reviewed every 1 week to check the response of the procedure and was continued for 4 weeks. The patient was instructed to maintain proper oral hygiene during the initial stage of treatment and advised to take a soft diet until the jawbone healed properly. Figure 31 shows the clinical picture of the patients after follow‐up and removal of the cap splint with normal occlusion. All three cases managed with different methods of approach according to the types of injuries they sustained showed a good clinical outcome. Alignment of mandible fracture, restoration of normal occlusion, esthetic, and phonetic improvement, which signifies that the clinical implications of this different appliance for the treatment modalities and approach for the management of these cases were effective and recommendable. Patient compliance was also acceptable with the application of these appliances for the management of certain types of pediatric dental trauma.

Figure 28(a, b) Ecchymosed with a fracture in a lingual site of the lower anterior region.

**Figure:**
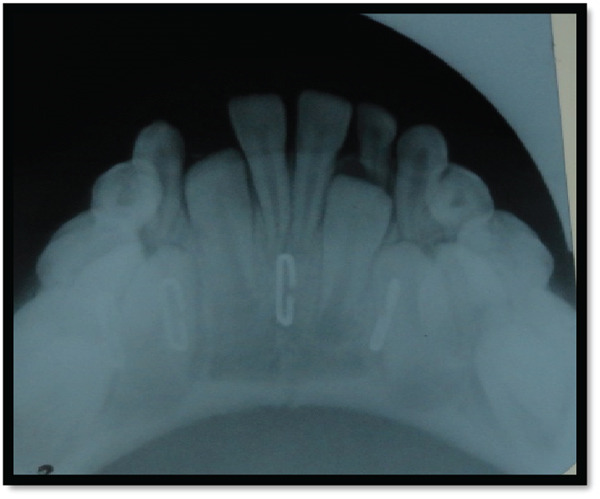
(a)

**Figure:**
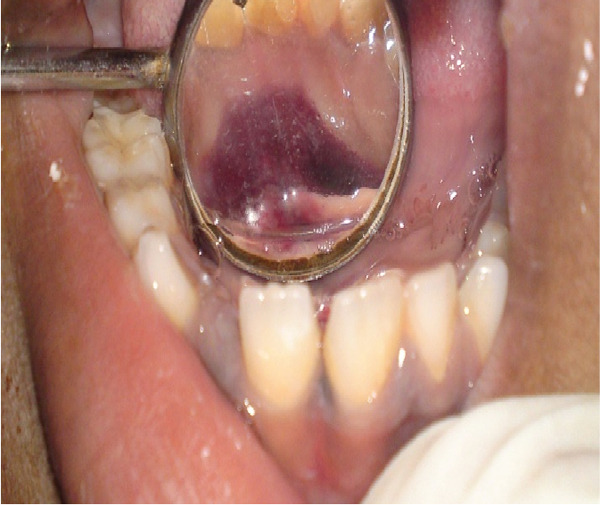
(b)

## 3. Discussion

Mandibular fractures are the most common (56%) of facial skeletal injuries in hospitalized pediatric trauma patients [19]. But their growing jaws and their anatomic variation, including their growing tooth bud, complicate and direct the choice of treatment plan, mostly in the case of displaced jaw fracture. But the goal of the treatment of these fractures is to restore the bone architecture in a stable position as less invasively as possible to restore function and esthetic impairment [20].

There are various techniques that have been utilized in the management of pediatric fractures, like tape muzzles, circumferential wiring, acrylic splints, percutaneous skeletal fixation, open reduction, resorbable plates, orthodontic resin, modified orthodontic brackets, rubber elastics in combination with orthodontic brackets, and nickel–titanium staples depending upon the minimally/severely displaced fractures [21]. But the treatment criteria should be minimally invasive with maximum benefit as far as possible, considering patient age, types of cases, etc.

The present case was with a slight displacement of the jaw with dearranged occlusion in both the male cases, where only tooth avulsion was present in the female case. So considering the types of trauma, the first female pediatric patient was managed with RPD, followed by the plastic surgery of the scar for restoration of functional, phonetic, and esthetic impairment of the oral cavity including facial skin. The two male pediatric patients were managed according to the types of injuries for which in Case No. 2 (PMMA) a self‐cure (PMMA) customised acrylic facial splint was fabricated and in Case No. 3 a customised self‐cure acrylic cap splint was fabricated to reduce the fracture and to restabilize an occlusion. There are some studies that have used modified closed‐cap splints for conservative management of minimally displaced pediatric mandibular fractures and came out with good results and mention that minimally displaced fractures can be managed conservatively by a modified cap splint compared to severely displaced fractures, which may require open reduction and rigid internal fixation [22]. There are also some studies done by them [23] in which they found that fabricated acrylic splints for conservative treatment of pediatric mandibular fracture are cost‐effective, easy to remove, less time‐consuming, and provide maximum stability during the healing period and minimal trauma to adjacent anatomic structures, making them more comfortable for young patients.

The present cases show that a customised acrylic splint, used for stabilizing and restoring normal occlusion, including functions of slight displacement of the mandible and occlusal arrangement in pediatric patients, was remarkable in terms of its clinical outcome. It can also be easily fabricated in any small dental setup where pediatric patients can be easily managed during mixed dentition. However, there are certain limitations with respect to acrylic splint fabrication, which requires alginate impressions that are cumbersome and time‐consuming to fabricate the splint.

## 4. Conclusion

The frequency of pediatric dental trauma increases with age due to various reasons, from infancy to adolescence. The management modalities also vary according to the types and severity of injuries they sustained that are challenging because of their growing age. There are various ways of managing trauma to restore normal occlusion and proper alignment of bone and permanent tooth buds without impairing growth and development. In cases of minimally displaced fractures, cap splints and other conservative methods are preferred. Two cases presented involve minimally displaced mandible fractures with slight occlusal dearrangement and one patient with dental trauma resulting in avulsion of permanent teeth. All cases were managed based on the specific needs and types of injuries sustained, with follow‐up until normal occlusion and bone alignment were achieved. The procedures used in these cases were simple, effective, cost‐effective, and painless, with minimal material requirements and no invasion of hard or soft tissue, making them easily implementable in small dental settings. This approach benefits patients by reducing nondisplaced mandible fractures, jawbone displacement, and occlusal dearrangement.

## Consent

We have taken consent for Case No. 2 only since the uploaded photographs of the patient’s full face along with the customised appliance were required to be shown for clear understanding. And the rest of the photograph only reveals the patient’s face without disclosing the patient’s identity.

## Conflicts of Interest

The authors declare no conflicts of interest.

## Author Contributions

**Lomtu Ronrang:** conceptualization, formal analysis, methodology, writing–original draft, review, and editing. **Alice Lyngdoh:** data curation and resources.

## Funding

We did not receive any funds from any agency or other sources in any form to conduct the present case series of the study.

## Data Availability

The authors have nothing to report.
